# Succinimide Derivatives as Acetylcholinesterase Inhibitors—In Silico and In Vitro Studies

**DOI:** 10.3390/cimb46060307

**Published:** 2024-05-22

**Authors:** Błażej Grodner, Dariusz Maciej Pisklak, Łukasz Szeleszczuk

**Affiliations:** 1 Chair and Department of Biochemistry and Pharmacogenomics, Faculty of Pharmacy, Medical University of Warsaw, 1 Banacha Str., 02-097 Warsaw, Poland; bgrodner@wum.edu.pl; 2 Department of Organic and Physical Chemistry, Faculty of Pharmacy, Medical University of Warsaw, 1 Banacha Str., 02-097 Warsaw, Poland; dpisklak@wum.edu.pl

**Keywords:** acetylcholine, acetylcholinesterase, Alzheimer’s disease, competitive inhibitors, enzyme activity, succinimide derivatives

## Abstract

We studied the effect of succinimide derivatives on acetylcholinesterase activity due to the interest in compounds that influence this enzyme’s activity, which could help treat memory issues more effectively. The following parameters were established for this purpose based on kinetic investigations of the enzyme in the presence of succinimide derivatives: the half-maximal inhibitory concentration, the maximum rate, the inhibition constant, and the Michaelis–Menten constant. Furthermore, computational analyses were performed to determine the energy required for succinimide derivatives to dock with the enzyme’s active site. The outcomes acquired in this manner demonstrated that all compounds inhibited acetylcholinesterase in a competitive manner. The values of the docking energy parameters corroborated the kinetic parameter values, which indicated discernible, albeit slight, variations in the inhibitory intensity among the various derivatives.

## 1. Introduction

The cholinergic enzyme acetylcholinesterase (AChE) is mostly located in postsynaptic neuromuscular junctions. Through hydrolysis, this enzyme converts the naturally occurring neurotransmitter acetylcholine (ACh) to acetic acid and choline [1,2]. As a neurotransmitter, ACh interacts with two different kinds of receptors, namely muscarinic receptors (belonging to the metabotropic receptor group) and nicotinic receptors (which belong to the ionotropic receptor group) [3]. It influences neurotransmitters in the peripheral, central, and autonomic nervous systems in a wide range of ways. In addition to participating in or modulating cognitive and memory functions, cholinergic neurons also affect attention, wakefulness, and reward. Additionally, muscle contraction requires acetylcholine as it functions as a neurotransmitter at the neuromuscular junction [4]. As already known, it can be inferred that the cholinergic system’s activity is mostly associated with the control of muscle contraction, which has a significant effect on memory, learning, and consciousness. As a result, the aging-related decrease in acetylcholine levels in the body is frequently accompanied by major alterations that cause a notable impairment in memory.

Alzheimer’s disease, one of the most prevalent types of dementia, has been shown to be strongly associated with low levels of acetylcholine in regions of the hippocampus. According to previous studies, it is thought that in patients with this illness, acetylcholine activity and the concentration of acetylcholine in the central nervous system drop by roughly 70%, which is linked to the degeneration of the neurons that make it [5].

A deficiency of acetylcholine in the body can potentially cause weakened muscle contractions and, in extreme cases, paralysis. Low levels of acetylcholine also contribute to the development of myasthenia gravis—an autoimmune muscle disease. Additionally, there are signs of double vision, breathing problems, difficulty swallowing, drooping eyelids, and general exhaustion [6].

However, a notable rise in the level of ACh is also caused by a considerable increase in the usage of irreversible AChE inhibitors, such as carbamate and organophosphorus chemicals, or battle gases (sarin, soman, and tabun) [7]. Miosis, eye pain, bronchospasm with cough and dyspnea, chest pain, and drooling are the initial signs and symptoms brought on by these inhibitors. Additional symptoms include a vomiting fit, diarrhea and abdominal pain, uncontrollable urination and bowel movements, and muscle tremors that are followed by convulsions and paralysis of the muscles. This finally results in respiratory failure brought on by the paralysis of the respiratory muscles, which causes problems that affect numerous bodily systems [8,9].

As a result, the control of AChE activity is crucial because changing the concentration of ACh in synaptic clefts may excessively stimulate or inhibit the activity of appropriate receptors (muscarinic and nicotinic), which may change a number of body functions [3,4,10].

Acetylcholine deficiency leads to the inhibition of the transmission of nerve impulses, resulting in muscle paralysis. Its low level leads to problems with memory and information processing. According to research, inadequate cholinergic activity and low levels of acetylcholine in the brain may contribute to cognitive loss and impaired memory in patients with dementia. Drugs that slow down dementia act by boosting the amount of acetylcholine in the brain [11]. Acetylcholine has also been linked to the development of or symptoms of some neurodegenerative illnesses, such as Alzheimer’s [12]. Alzheimer’s disease causes the slow deterioration and destruction of “cholinergic” neurons, which are brain cells that primarily utilize acetylcholine. In addition, as the condition advances, key molecules known as acetylcholine transporters may become compromised. These molecules are crucial for delivering acetylcholine into neurons; therefore, weakening them might make it more difficult for acetylcholine to complete its regular tasks in the brain. Thus, these two essential acetylcholine-related pathways are expected to considerably contribute to some of the defining cognitive symptoms of these illnesses [13,14]. Many (but not all) of the drugs used to treat Alzheimer’s disease are acetylcholinesterase inhibitors. As the name implies, these medications suppress the enzyme acetylcholinesterase, which is responsible for the breakdown of the neurotransmitter acetylcholine in the brain. Inhibiting these enzymes may result in an overall increase in acetylcholine levels and activity, perhaps compensating for the loss of cholinergic neurons seen in Alzheimer’s disease [13,14]. Acetylcholinesterase inhibitors, such as galantamine and donepezil, are commonly used to treat Alzheimer’s disease. Their efficacy in reducing certain cognitive symptoms of the disease is primarily attributed to their capacity to stimulate acetylcholine activity in the brain [15,16,17,18,19]. ACh has a significant impact on the induction (formation) of REM sleep, as evidenced by the significantly larger drop in ACh levels in particular brain regions during awake or NREM sleep compared to REM sleep [20]. Therefore, a shortage in acetylcholine may result in problems with sleep by causing the so-called REM phase to either completely disappear or cease to exist. Dreams occur during this phase.

A lack of proper regulation of AChE and the resulting disturbances in acetylcholine levels also have a negative impact on the functioning of the central nervous system and cognitive functions, as well as in the development of some mental diseases, such as schizophrenia and other psychotic states [21,22,23].

Compounds that are activators or inhibitors are utilized in the regulation of acetylcholinesterase. Chemicals from different chemical groups make up the chemicals that accelerate the breakdown of ACh and the compounds that activate AChE. While some of them are more complex molecules [24,25,26], others are simple ions such as Mg^2+^, Ca^2+^, Mn^2+^, and Na^+^ [3]. All activators are common in that they activate acetylcholinesterase, which causes an excessive amount of acetylcholine to be broken down. This can result in a variety of symptoms, including those previously described [4,27,28,29,30,31,32,33,34], such as constipation, gastroparesis, memory issues, speaking difficulties, learning difficulties, dry mouth, dry eyes, orthostatic hypotension, low muscle tone, depression, fast heart rate, chronic inflammation, and emotional instability.

AChE inhibitors, on the other hand, have the ability to decrease AChE activity, which, in turn, results in less ACh being broken down, a rise in ACh content in the synaptic cleft, and an overstimulation of cholinergic receptors. This finally results in respiratory failure due to paralysis of the respiratory muscles, which, in turn, causes many of the abnormalities of various biological functions already discussed [35]. In addition to the medications listed above, the class of chemicals known as AChE inhibitors also includes a variety of additional medicines, including analogues of 9-amino-1,2,3,4-tetrahydroacridine [36].

It is also commonly known that a variety of factors influence the maintenance of an appropriate level of ACh in the body’s various structures. Appropriate AChE activity is one of them. The second one contains a wide range of chemicals.

AChE is hypothesized to have both enzymatic and non-enzymatic action, impacting numerous physiological pathways such as cell adhesion, neurite outgrowth, and the creation of amyloid fibrils. Some non-catalytic activities are attributed to the peripheral anionic site (PAS), which is situated outside of the AChE narrow gorge and functions as the secondary binding site for ACh and quaternary ligands in the absence of enzyme activity. For instance, a hypothesis concerning the role of amyloid β (Aβ) in Alzheimer’s disease (AD) contends that Aβ deposition is a key pathogenic indicator of the onset and development of AD. A study by Inestrosa et al. [37] identified AChE as a molecular chaperone that uses the peripheral anionic site (PAS) to speed up the assembly of Aβ into oligomers and fibrils in amyloidosis.

Therefore, AChE inhibitors that block peripheral locations may function as disease-modifying drugs by postponing the development of amyloid plaque. Because of this, it was essential to forecast the potential interaction location of the chemicals under study and determine whether they would bind to the PAS of AChE or preferentially interact with the active site of an enzyme.

Reversible AChE inhibitors, such as galantamine, rivastigmine, and donepezil, are frequently used in medicine because they block acetylcholinesterase activity while also preventing ACh from being broken down in the brain. This increases acetylcholine concentration and improves nerve cell communication [8,38,39,40,41,42]. These medications, which reduce symptoms by preventing ACh from being broken down, are crucial in the treatment of dementia and neurodegenerative symptoms associated with Alzheimer’s disease. They also aid in keeping the concentration of ACh at the proper amount [43,44,45,46].

**Figure 1:**
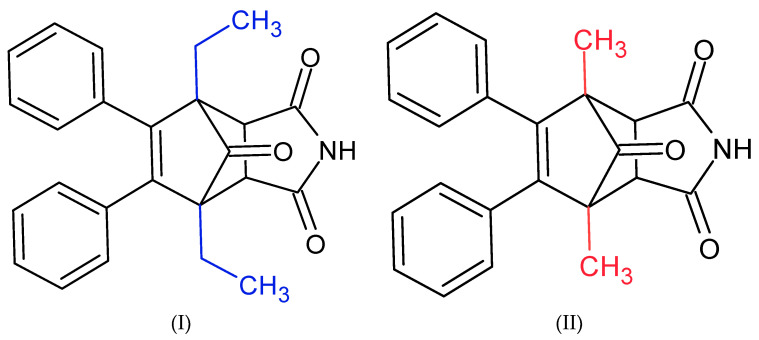
Structures of succinimide derivatives (I) and (II).

In this work, we examined how succinimide derivatives (Figure 1) affect acetylcholinesterase activity and the potential for interactions with the enzyme’s active site.

## 2. Materials and Methods

### 2.1. Reagents and Chemicals

Acetylcholinesterase human (EC. 3.1.1.7, AChE), acetylthiocholine iodide (ACthI), 5,5′-dithiobis (2-nitrobenzoic) acid (DTNB), and 5-thio-2-nitrobenzoic acid (NTB) were obtained from Sigma-Aldrich (Poznań, Poland). Diethyl and dimethyl succinimide derivatives were purchased from our laboratory (Medical University of Warsaw). The purities of the reference compounds were found to be greater than 98%.

### 2.2. Instrumentation

A JASCO V-730 PC UV-VIS spectrophotometer with a range of 190–1100 nm (JASCO Corporation, Tokyo, Japan) was used to perform kinetic measurements of enzymatic reactions.

### 2.3. Preparation of Stock and Working Standards

Diethyl (I) and dimethyl (II) succinimide e derivatives were produced as primary stock standard solutions in deionized water (concentration of each = 50 mM). Additionally, primary stock standard solutions for ACthI were made independently using deionized water. A concentration of 46.00 mM was achieved by dissolving 332.5 mg of ACthI in 25 mL of phosphate buffer (100 mM, pH 7.5). After diluting the produced ACthI solution with 100 mM of phosphate buffer, mixed working standard solutions with concentrations of 34.50, 23.00, 11.50, 5.75, 2.88, 1.44, 0.72, and 0.36 mM were obtained.

### 2.4. Sample Preparation

After dissolving the AChE in 100 mM of phosphate buffer (pH 7.5), it was separated into multiple pieces and kept at −20 °C for storage. Every day, one aliquot was thawed, and the AChE activity was monitored. Just before usage, the appropriate concentrations of DTNB and ACthI working solutions were made by diluting them with phosphate buffer.

**Figure 2:**
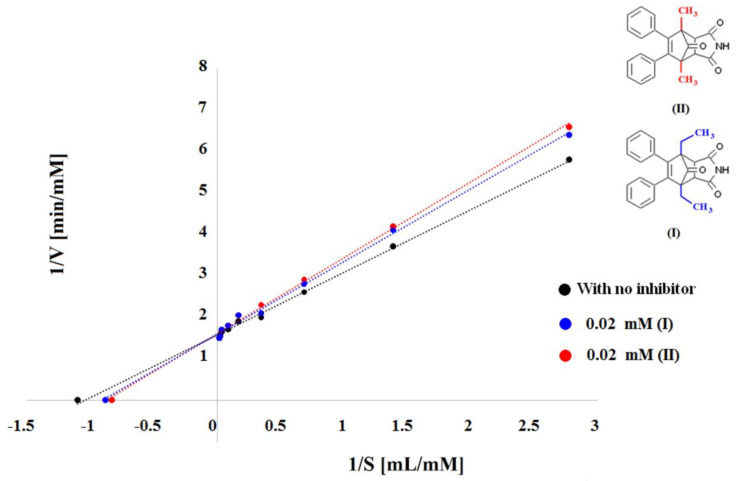
Lineweaver–Burk plots for systems with inhibitors (I) and (II) at a concentration of 0.02 mM.

The approach by Ellman et al. [27] and the slightly modified process by Kaizer R.R. et al. [47] were used to determine acetylcholinesterase activity [48,49,50,51]. In a nutshell, 1.7 mL of pH 7.5 phosphate buffer (100 mM concentration) containing 0.36–46.00 mM of ACthI, 0.02 mL of inhibitor solutions (I) and (II), 0.1 mL of 4 mM 5,5′-dithio-bis-2-nitrobenzoic acid (DTNB), and 0.08 mL of water were added to nine test tubes; 1.8 mL of phosphate buffer (100 mM concentration, pH 7.5) containing ACthI (0.36 mM concentration), 0.1 mL of 4 mM DTNB, 0.02 mL of (I) and (II) inhibitor solution, and 0.08 mL of H_2_O were added to another tube (zero sample, tube 10). The tubes holding the solutions were pre-incubated at 25 °C for two minutes. Subsequently, 0.1 mL of 50 mM AChE was added to tubes 1 through 9, one minute apart. Ten samples were incubated at 25 °C for three minutes. The analytical samples were placed in the analytical cuvette after incubation. At 412 nm, the resultant product’s color intensity was measured. Table 1 in the Section 2.4 and Figure 2 on the Lineweaver–Burk coordinate system display the findings from determining the inhibition type using the following equation:

1/V = K_m_/V_max_ [S] + 1/V_max_

### 2.5. Docking Studies

The PyRx docking tool [52] was used in molecular docking investigations via the Autodock VINA program [53,54] in order to determine the possible binding site of the compounds under analysis and assess their interaction with the AChE enzyme. The choice of the docking program resulted from the fact that in previous works, the authors had shown that the AutoDock Vina package was best able to reproduce the bioactive conformation of model compounds when docking to the ACHE enzyme [55]. For the purpose of docking, structures developed for room-temperature X-ray diffraction studies were chosen in an attempt to closely mimic the native form of the enzyme. Two of these human AChE enzyme structures, identified by PDB codes 6O4W and 6O4X, are stored in the RCSB Protein Data Bank database [56] and deposited with resolutions of 2.35 Å and 2.3 Å, respectively. Both structures deposited in the PDB database were homodimers. One subunit was removed from the structure (including the ligand and water molecules); therefore, only a single subunit defined as the A chain was used for docking. The model inhibitors donepezil and 9-aminoacridine were used to crystallize these structures, and the resulting resolutions were 2.35 and 2.30, respectively. The PyMOL software (the PyMOL Molecular Graphics System, Version 2.0, Schrödinger, LLC, New York, NY, USA) was used to analyze and prepare for docking investigations for the three-dimensional (3D) structures (PDB format) of both AChE structures [57]. The gorge of the peripheral anionic site and the catalytic center of the enzyme were both contained in a 25 × 25 × 25 grid box pocket. Based on the presence of amino acids designated as PAS (Tyr 72, Tyr 74, Tyr 124, Trp 286, Phe 297, and Tyr 341) and active sites (Ser 203, Glu 334, and His 447), both sites were identified.

The Lamarckian genetic algorithm was utilized to search for the conformations using the following docking parameters: a population size of 150 dockings, a maximum number of generations of 27,000, a maximum energy evaluation of 25 million, 50 docking runs, and random initial positions and conformations [58].

Both compounds (I and II) were chosen as ligands, and donepezil and 9-aminoacridine’s structures were also considered as model inhibitors of AChE. The docking mechanism did not take advantage of any limitations. In order to optimize the conformational binding analysis, the exhaustive value of 40 was employed. Based on the binding affinity values (kcal/mol) and binding interaction patterns (hydrogen, hydrophobicity, and electrostatic), the produced docked complexes were chosen, and their conformations were examined for higher scores. All docked complexes were shown graphically with the help of the Discovery studio visualizer version 4.0 (BIOVIA, San Diego, CA, USA).

### 2.6. Evaluation of ADMET Properties

For both compounds, after converting the structures to the SMILES format, ADMET parameters were also estimated using the ADMETlab server (https://admetlab3.scbdd.com, accessed on 14 May 2024) [59].

## 3. Results

In order to ascertain whether diethyl (I) and dimethyl (II) succinimide derivatives block AChE, the inhibition of enzymatic activity was examined in this work.

### 3.1. Experimental

In this work, acetylthiocholine (ACh), 5-thio-2-nitrobenzoic acid (NTB), 5,5′-dithio-bis-2-nitrobenzoic acid (DTNB), acetylcholinesterase (AChE), and inhibitors (I) and (II) of acetylcholinester-ase were present when we determined the main enzyme reaction product, 4,4-dithio-bis-nitrobenzoic acid (NBA), which was determined using Ellman’s method. Nine reaction substrate (ACh) concentrations (0.36, 0.72, 1.44, 2.88, 5.75, 11.50, 23.00, 34.50, and 46.00 mM) were used to examine the fundamental enzymatic activity in the presence of the enzyme (AChE). Enzymatic kinetics research was conducted in a system containing progressively increasing quantities of the substrate at a constant concentration (0.02 mM) of inhibitors (I) and (II) to assess the effect of the inhibition of compounds (I) and (II). In the absence of inhibitors, the correlation coefficient for ACh was shown to be 0.9983, and the curve’s slope was 1.509. Compounds (I) and (II) had correlation coefficients of 0.9972 and 0.9975 at a concentration of 0.02 mM, and their corresponding curve slopes were 1.754 and 1.829 (Table 1 and Figure 2).

Kinetic studies were carried out in order to learn more about the AChE inhibition mechanism using (I) and (II) succinimide derivatives. Lineweaver–Burk plots (Figure 2) were created based on the inhibition data collected during steady-state conditions. These plots illustrate the reciprocal relationship between the reaction substrate concentration (acetylthiocholine) and the reciprocal of the reaction rate.

### 3.2. Analysis

Plots of straight lines with various slope angles (Km/Vmax) for inhibitors (I) and (II) obtained at the concentration of 0.02 mM intersected at one point on the y axis, which corresponded to the reaction rate’s reciprocal value (Vmax). Reversible competitive AChE inhibition suggests such a mechanism. A competitive form of inhibition is also suggested by the computed Km and Vmax values for these systems (Table 2).

First, it could be demonstrated that compound (II) was a stronger competitive inhibitor of AChE based on differences in Km values between individual compounds. This was demonstrated by compound (II) having the highest Km values for the succinimide derivative (1.11 mM/mL) compared to derivative (I), which had a Km value of 1.15 mM/mL.

The next value used to calculate the inhibition force was the angles of the Lineweaver–Burk straight lines. In this case, too, derivative (II) proved to be a stronger AChE inhibitor than derivative (I) because the values of these angles were higher for inhibitor (II) (61.34°) than for inhibitor (I) (60.31°) (Table 2 and Figure 2).

Next, for the inhibitors (I) and (II), the inhibition constants (Ki) were computed (Table 2). In comparison to inhibitor (I), the Ki values show that inhibitor (II) binds to the AChE active site more strongly. Thus, it may be said that whilst compound (I) was a weaker inhibitor of AChE, compound (II) was a stronger inhibitor of this enzyme.

**Figure 3:**
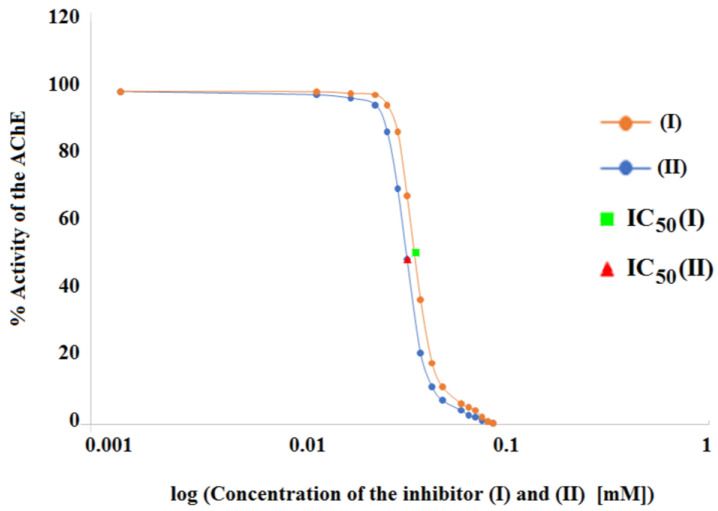
Graphical determination of IC_50_ value for (I) ■ and (II) ▲ acetylcholinesterase inhibitors.

The half-maximum inhibitory concentration (IC_50_) values, or the number of individual derivatives (I) and (II) required to inhibit AChE by 50%, were determined in order to gather further information on the potency of AChE inhibition by compounds (I) and (II) (Table 2 and Figure 3).

Compound (II) produced AChE inhibitory action at a lower concentration than compound (I), as can be shown by comparing their IC_50_ values of 0.029 mM and 0.031 mM. Thus, it may be concluded that compound (II) was the more effective AChE inhibitor in this instance as well, surpassing inhibitor (I).

This study revealed that the type of substituent (methyl or ethyl) present in the primary chemical molecule of compounds (I) and (II) determines the inhibitory potency and affinity strength of two derivatives. This study’s kinetic parameter values, which show good correlation with the derivatives’ docking studies to the enzyme’s active site, further support the idea that derivative (II) is a more potent AChE inhibitor (Table 3).

We also examined the potential for competing inhibitor compounds to interact with the active site of acetylcholinesterase, taking into account the enzyme’s known spatial structure. AChE’s active site, which is made up of the catalytic site and the peripheral anionic site (PAS), is situated at the bottom of a 20 Å long gorge. The catalytic trio (Ser 203, Glu 334, and His 447) makes up the catalytic site. The five residues (Tyr 72, Asp 74, Tyr 124, Trp 286, and Tyr 341) grouped around the entrance to the gorge of the active site constitute the peripheral anionic site, which binds the positively charged component of the ligand with the amino acid Tyr 86 of an enzyme, which is located at the entrance to the active site [58].

**Figure 4:**
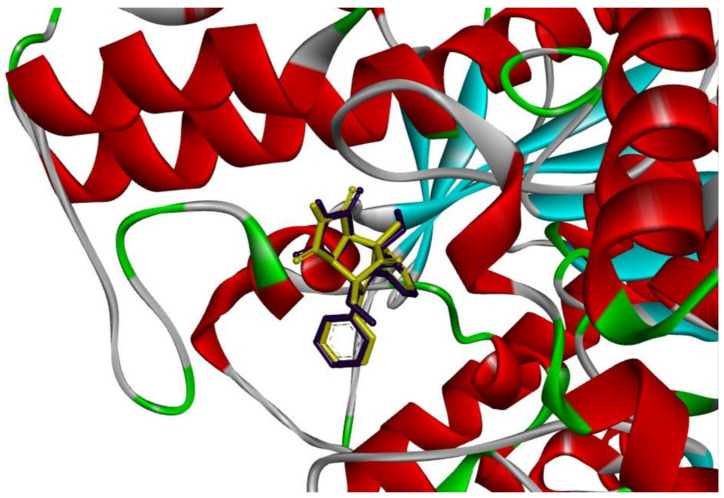
Binding mode of compounds I (purple) and II (yellow) in active site of AChE enzyme. For visualization, Discovery Studio visualizer version 4.0 (BIOVIA, San Diego, CA, USA) was used.

Molecular docking techniques were applied to determine the compounds’ probable binding sites as well as potential interactions that could stabilize the protein–ligand relationship. The fundamental inquiry was how the investigated molecules might attach to the AChE active site and whether they could reach the center of the active site and engage with the catalytic triad due to the presence of a sterically enlarged imide moiety. Two human acetylcholinesterase crystal structures were chosen for docking studies: one crystallized with the model inhibitor donepezil (6o4w), which binds to both sites, and the other crystallized with 9-aminoacridine (6o4x), which only binds to PAS (Figure 4).

Using the Autodock Vina tool, both drugs (I and II) as well as donepezil and 9-aminoacridine as model inhibitors were docked in the crystallographic structure of the AChE active site (Table 4).

In accordance with the experimental data, the docking results demonstrate that the scoring function values in both scenarios were marginally higher for the evaluation of compound II’s binding than compound I. Simultaneously, docking to the 6O4W crystal structure—which was formed using donepezil—produced somewhat higher values. The values obtained for compounds I and II were comparable to those obtained for 9-aminoacridine, but significantly lower than those obtained for donepezil (which was in line with the experimental results).

A subsequent examination of the binding interaction revealed that, similar to 9-aminocradine, the investigated compounds engage more with the peripheral anionic site (PAS) at the opening to the active site gorge (Figure 4). They are unable to enter the AChE active site gorge and interact directly with the catalytic site of the enzyme due to the existence of a spatially extended imide substituent and the absence of an elongated chain (as in donepezil). The bioactive conformations and intramolecular interactions in the ligand–enzyme complex are comparable for both drugs despite the modest differences in their structures. Acridine-based pharmacophore 1,2 has been linked to numerous biological activities, particularly in the inhibition of cholinesterase activity, for the treatment of Alzheimer’s disease (AD).

**Figure 5:**
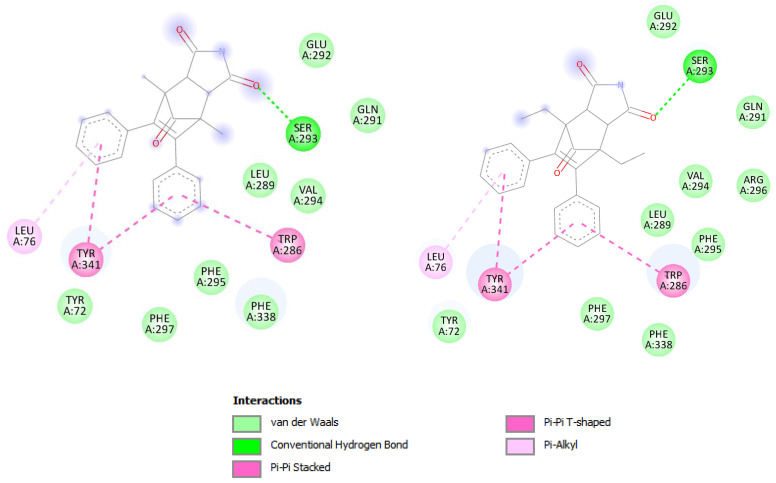
Representation of interactions between PAS of AChE enzyme and docked compounds I and II.

According to docking studies, the investigated compounds interact with the Trp 286 and Thr 341 of the PAS of the AChE enzyme through the alkyl-π bond with LEU 76 and the π-π dispersion force. In addition, the hydrogen bond with the amino acid SER 293 may have an impact on the stability of the protein–ligand interaction because of the imide substituent that is present (Figure 5).

ADMETlab 3.0 was employed to calculate descriptors associated with absorption, distribution, metabolism, toxicity, excretion, and the human intestinal absorption of (I) and (II) [60]. Those results can be found in Supplementary Files S1 and S2, respectively. The two studied compounds were characterized by similar ADMET properties.

## 4. Discussion

After comparing the inhibition potency (IC_50_) of succinimide derivatives (I) and (II) to model compounds like Physostigmine, donepezil, and rivastigmine [7], it can be inferred that succinimide derivatives occupy an intermediate position among the three AChE inhibitors that are most frequently used in medicine.

Both succinimide compounds have IC_50_ values between 29,000 and 31,000 µM, which indicates that they are 1047–1448 times less effective AChE inhibitors than donepezil (IC_50_ = 0.027 µM). The inhibitory activities of these derivatives are only 161–172 times weaker than that of Physostigmine (IC_50_ = 0.18 µM). Nonetheless, succinimide compounds seem to be 2.4–2.3 times more powerful AChE inhibitors than rivastigmine, whose IC_50_ value is 71,000 µM.

Additionally, we compared the values of the succinimide derivatives’ affinity strength (Ki) with those of donepezil, Physostigmine, and rivastigmine’s affinity to AChE in this study.

The Ki values range from 1.5 × 10^−5^ M to 1.5 × 10^−3^ M for rivastigmine, from 3 × 10^−8^ M to 9.7 × 10^−7^ M for Physostigmine, and from 1.4 × 10^−10^ M to 5.6 × 10^−6^ M for donepezil [61]. In contrast, our analysis yielded Ki values of 6.38 × 10^−3^ M and 5.13 × 10^−3^ M for succinimide derivatives (I) and (II), respectively.

Thus, by comparing the Ki values for succinimide derivatives with those for donepezil, Physostigmine, and rivastigmine, it is possible to determine that the affinity strength of aminoalkanol derivatives for AChE is from 3.4 to 4.3 × 10^3^ times weaker than rivastigmine, from 5.3 × 10^3^ to 2.1 × 10^5^ times weaker than donepezil, and from 3.7 × 10^7^ to 9.2 × 10^2^ times weaker than donepezil.

This study’s findings demonstrate that succinimide derivatives with methyl substituents inhibited AChE more than those with ethyl substituents. The obtained data indicate that the potency of AChE inhibition is clearly influenced by the type and size of substituents.

As is widely known, the half maximal inhibitory concentration (IC_50_) is a measure of the potency of a substance in inhibiting a specific biological or biochemical function. The IC_50_ value is a quantitative measure that indicates how much of a particular inhibitory substance (e.g., drug) is needed to inhibit, in vitro, a given biological process or biological component by 50%. Succinimide compounds seem to be 2.4–2.3 times more powerful AChE inhibitors than rivastigmine, which is a commonly used drug for Alzheimer’s disease and has an IC_50_ value of 71,000 µM.

## 5. Conclusions

We tried to examine the impact of succinimide e derivatives on AChE activity because they were not identified as inhibitors and had not been documented in the literature as of yet. Our research shows that a spectrophotometric analysis, in an in vitro drug metabolic system, can be a straightforward method for evaluating AChE inhibition.

AChE was inhibited in a competitive manner by both succinimide derivatives. Derivative (II) was found to be a more powerful AChE inhibitor than derivative (I) based on the Km, Vmax, Ki, and IC_50_ values. The observed results imply that the type of substituent present in the compound’s primary structure determines the inhibitory potency. Since there are only small variations in the IC_50_ values, it is possible to conclude that all of the substances under study inhibit acetylcholinesterase to a similar degree. But the technique we created made it possible for us to record these minute variations.

Furthermore, docking studies indicate that because of the sterically extended substituent, the investigated compounds bind with the PAS rather than entering the catalytic pocket.

## Figures and Tables

**Table 1:** The regression equation and quantification for acetylthiocholine for 6 replicates for each sample (*n* = 6) in the concentration range of 0.36–46.00 mM in the presence of inhibitors (I) and (II) at a concentration of 0.02 mM.

Concentration of Inhibitors (mM)	Linearity Range of Substrate (Acetylthiocholine) (mM)	R^2^	RSD (%)	Regression Equation	Standard Deviation	
					Slope	Intercept
0	0.36–46.00	0.9983	3.01	y = 1.509x + 1.572	±0.054	±0.031
(I) 0.02	0.36–46.00	0.9972	3.54	y = 1.754x + 1.579	±0.077	±0.065
(II) 0.02	0.36–46.00	0.9975	2.36	y = 1.829x + 1.582	±0.072	±0.059

RSD, relative standard deviation.

**Table 2:** The analytical data describing the effects of acetylcholinesterase inhibition by succinimide derivatives (I) and (II) and the regression equation for acetylthiocholine for 6 replicates for each sample (*n* = 6) at the concentration range of 0.36–46.00 mM in the presence of inhibitors (I) and (II) at a concentration of 0.02 mM.

**Compound (I)**				
**Concentration (mM)**	**Straight Line Equation**	**R^2^**	**Tilt Angle (°)**	
(I) 0.00	y = 0.3680x + 0.3828	0.9983 ± 0.0014	56.47 ± 0.08	
(I) 0.02	y = 0.3917x + 0.4505	0.9972 ± 0.0021	60.31 ± 0.11	
**Concentration (mM)**	**Km (mM/mL)**	**Vmax (mM/min)**	**Ki (mM)**	**IC_50_ (mM)**
(I) 0.00	0.96 ± 0.05	0.63 ± 0.04	-	-
(I) 0.02	1.15 ± 0.07	0.63 ± 0.06	6.38 ± 0.06	0.031 ± 0.003
**Compound (II)**				
**Concentration (mM)**	**Straight Line Equation**	**R^2^**	**Tilt Angle (°)**	
(II) 0.00	y = 1.509x + 1.572	0.9983 ± 0.0014	56.47 ± 0.08	
(II) 0.02	y = 1.830x + 1.595	0.9975 ± 0.0019	61.34 ± 0.06	
**Concentration (mM)**	**Km (mM/mL)**	**Vmax (mM/min)**	**Ki (mM)**	**IC_50_ (mM)**
(II) 0.00	0.96 ± 0.05	0.63 ± 0.04	-	-
(II) 0.02	1.11 ± 0.03	0.63 ± 0.04	5.13 ± 0.04	0.029 ± 0.002

**Table 3:** The effect of the type of substituents occurring in the main structure of succinimide derivatives (I) and (II) on the inhibitory potency (IC_50_) and affinity strength (Ki) of AChE.

Compound	R_1_	R_2_	Ki [mM]	IC_50_ [mM]	Docking Score [kcal/mol]
Derivative (I)	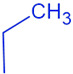	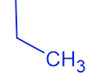	6.38	0.031	−8.9
Derivative (II)	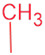	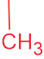	5.13	0.029	−9.8

**Table 4:** Docking results.

	Docking Score [kcal/mol] 6O4W Structure	Docking Score [kcal/mol] 6O4X Structure
I ethyl	−8.9	−8.3
II methyl	−9.8	−9.0
Donepezil	−12.6	−9.2
9-aminoacridine	−8.9	−9.2

## Data Availability

The data can be obtained from the corresponding author (B.G.) by email.

## Supplementary Materials

The following supporting information can be downloaded at https://www.mdpi.com/article/10.3390/cimb46060307/s1, File S1: ADMET properties of II; File S2: ADMET properties of I.
